# Improved Distance Queries and Cycle Counting by Frobenius Normal Form

**DOI:** 10.1007/s00224-018-9894-x

**Published:** 2018-11-21

**Authors:** Piotr Sankowski, Karol Węgrzycki

**Affiliations:** Institute of Informatics, University of Warsaw, Warsaw, Poland

**Keywords:** Graph algorithms, Frobenius normal form, All-nodes shortest cycles

## Abstract

Consider an unweighted, directed graph *G* with the diameter *D*. In this paper, we introduce the framework for counting cycles and walks of given length in matrix multiplication time $\widetilde {O}(n^{\omega })$. The framework is based on the fast decomposition into Frobenius normal form and the Hankel matrix-vector multiplication. It allows us to solve the All-Nodes Shortest Cycles, All-Pairs All Walks problems efficiently and also give some improvement upon distance queries in unweighted graphs.

## Introduction

The *All-Pairs Shortest Paths* (APSP) problem asks to find distances between all pairs of vertices in a graph. For a directed graphs with weights in $\mathbb {R}$, there is a classical *O*(*n*^3^) time algorithm [11, 29]. Currently best upper bound for this problem is due to [30] who showed an $O\left (\frac {n^{3}}{2^{{\Omega }(\log n)^{0.5}}}\right )$ algorithm. It is asymptotically faster than *O*(*n*^3^/log*cn*) for any *c* > 0 (see survey [6] for earlier algorithms). Showing any algorithm that would work in *O*(*n*^3−*𝜖*^) time for some *𝜖* > 0 is a major open problem [30].

If we consider unweighted, directed graphs there are subcubic algorithms that exploit fast matrix multiplication. For the undirected graph [24] presented the optimal $\widetilde {O}(n^{\omega })$ time algorithm, where *ω* < 2.373 is the matrix multiplication exponent [16]. For the directed case [35] presented an *O*(*n*^2.575^) time algorithm that is based on the fast rectangular matrix multiplication. Moreover, if we are interested in small integer weights from the set {−*M*,…,*M*} we have *O*(*M*^0.68^*n*^2.575^) algorithm [35].

Because APSP in undirected graphs can be solved in $\widetilde {O}(n^{\omega })$, diameter, radius, shortest cycle, etc. can be determined in $\widetilde {O}(n^{\omega })$ time as well. It is surprising that for a directed case, where merely *O*(*n*^2.575^) APSP is known there are also $\widetilde {O}(n^{\omega })$ algorithms for determining these properties. After a long line of improvements [9] showed an $\widetilde {O}(M n^{\omega })$ time algorithms for finding minimum weight perfect matching, shortest cycle, diameter and radius (some of these results were already known [21]). Also, [9] showed an application of their techniques that improves upon [31] $\widetilde {O}(M n^{\omega } t)$ time algorithm for the following problem: *determine the set of vertices that lie on some cycle of length at mostt*. Cygan et al. [9] managed to solve this problem in $\widetilde {O}(M n^{\omega })$ time using Baur-Strassen’s theorem.

All of these algorithm are effective merely in the case of a dense graphs. For graphs with the small number of edges there are more efficient algorithms (e.g., APSP in $\widetilde {O}(|V||E|)$ time [27]). But these algorithms are Θ(*n*^3^) when |*E*| = Θ(*n*^2^).

### Related Work

#### Distance Queries

Yuster and Zwick [33] considered the weighted, directed graphs with weights in {−*M*,…,*M*}. They showed an algorithm that needs $\widetilde {O}(M n^{\omega })$ preprocessing time. After preprocessing each distance *δ*(*u*, *v*) in the graph can be computed exactly in *O*(*n*) query time. In the special case *M* = 1 they showed $\widetilde {O}(n^{\omega })$ algorithm that solves *Single Source Shortest Paths* (SSSP). This is the best known algorithm for a dense, weighted graph.

We will match their bounds (up to the polylogarithmic factors) using Frobenius normal form. Next we will extend that approach so it will return more information about a graph in the same query/preprocessing time.

#### Counting Cycles

For a given graph *G* and *k* determining whether *G* contains a simple cycle of length exactly *k* is NP-hard (in particular determining whether a graph contains a Hamiltonian cycle is NP-complete). However, if we fix *k* to be a constant this problem can be solved in polynomial time.

Alon et al. [4] introduced a color coding technique. For a fixed *k* if a graph *G*(*V*, *E*) contains a simple cycle of size exactly *k* then such a cycle can be found in $\widetilde {O}(|V|^{\omega })$ time. Unfortunately, their algorithm depends exponentially 2^*O*(*k*)^ on the length of the cycle and in consequence is inapplicable for large *k*. In the next years, [5] showed (using a different technique) that for *k* ≤ 7 one can count the number of cycles of length exactly *k* in a graph in $\widetilde {O}(|V|^{\omega })$ time. In [32] it is shown that for any even *k*, cycles of length *k* can be found in *O*(|*V* |^2^) time in undirected graphs (if they contain such a cycle). Alon et al. [5] showed more methods that depend solely on a number of edges in a graph. For example for odd *k* they showed $O\left (E^{2-\frac {2}{k + 1}}\right )$ algorithm for finding cycles of length *k*. However, for dense graphs these results are worse than [4].

From the other hand, to detect whether a non-simple cycle of length exactly *k* exists one can use the folklore algorithm. It starts by taking the adjacency matrix *A* of a graph *G*. Subsequently, in $\widetilde {O}(n^{\omega })$ time compute *A*^*k*^ by repeated squaring. If Tr [*A*^*k*^] > 0 then there exists a non-simple cycle of length *k*.^1^

Yuster [31] considered the following problem: *for every vertex in a graph find a shortest cycle that contains it*. He called this problem *All-Nodes Shortest Cycle* (ANSC). He showed a randomized algorithm that solves ANSC for undirected graphs with weights {1,…,*M*} in $\widetilde {O}(\sqrt {M} n^{(\omega + 3)/2})$ time. He noted that for simple digraphs (directed graphs with no anti-parallel edges) it reduces to All-Pairs Shortest Path problem. The fastest known APSP algorithm for unweighted, directed graphs runs in *O*(*n*^2.575^) due to [35]. Here, we will show how to solve ANSC in $\widetilde {O}(n^{\omega })$ for general, unweighted, directed graphs. Unfortunately, our techniques will allow us merely to find the length of such a cycle. But we can return the set of points, that lie on some cycle of a given length. Independently to our work [3] proved that ANSC can be solved in $\widetilde {O}(n^{\omega })$ for unweighted, undirected graphs using a completely different technique.

Yuster [31] also considered following problem: *given a graph and an integert*. *LetS*(*k*) *denote the set of all vertices lying in a cycle of length*≤ *k*. *DetermineS*(*t*). He considered directed graphs with weights in {−*M*,…,*M*} and showed $\widetilde {O}(M n^{\omega } t)$ algorithm. Recently, [9] improved his algorithm. They showed that for a fixed *t* ∈ [0,*nM*] the set *S*(*t*) can be computed in $\widetilde {O}(M n^{\omega })$ randomized time. We show, that for unweighted (*M* = 1) directed graphs we can compute sets *S*(1),*S*(2),…,*S*(*D*) in $\widetilde {O}(n^{\omega })$ time with high probability.

## Preliminaries

Let *T*(*n*) be the minimal number of algebraic operations needed to compute the product of *n* × *n* matrix by an *n* × *n* matrix. We say that *ω* is the exponent of square matrix multiplication. For now the best known upper bound on *ω* is due to [16]:

### **Theorem 1**

[16] *For every𝜖* > 0,*T*(*n*) < *O*(*n*^*ω* + *𝜖*^) *whereω* < 2.37287*.*

In this paper, we will omit *𝜖* in definition and will assume that *O*(*n*^*ω*^) operations are needed to multiply two matrices. The best lower bound for the exponent of matrix multiplication is *ω* ≥ 2. For convenience in this paper we will assume that *ω* > 2. The $\widetilde {O}$ notation hides polylogarithmic factors in complexity. We will use it to emphasize that all our algorithms need polylogarithmic number of calls to the fast matrix multiplication algorithm.

In this paper we will consider the Word RAM model of computation with a word size *O*(log *n*). The $\mathbb {F}$ denotes a small finite field and often we will assume this field to be $\mathbb {Z}_{p}$ for a prime number *p* with *O*(log *n*) bits. Note, that in Word RAM model arithmetic operations in $\mathbb {Z}_{p}$ can be done in constant time. In some situations we will need to stress that we want to work on larger integers. We will use *W* to denote the upper bound on the largest number in the field. In this case the arithmetic operations in Word RAM model take *O*(log *W*) time.

For matrices $A \in \mathbb {F}^{n\times k}$ and $B \in \mathbb {F}^{n \times l}$ the $A \oplus B \in \mathbb {F}^{n \times (k+l)}$ is the concatenation of their columns. $C_{a,b} \in \mathbb {F}^{n\times (b-a)}$ denotes a matrix constructed by concatenating columns *c*_*a*_,*c*_*a*+ 1_,…,*c*_*b*_ of matrix $C \in \mathbb {F}^{n \times m}$.

In this paper we will consider the unweighted, directed graph *G* unless stated otherwise. We will denote by *V* (*G*) and *E*(*G*) the sets of vertices and edges of graph *G*. The adjacency matrix *A*(*G*) ∈{0,1}^*n*×*n*^ of a directed graph *G* with *n* vertices is defined as:

$$A(G)_{i,j} = \left\{\begin{array}{lll} 1 & \text{if} i \rightarrow j \in E(G) \\ 0 & \text{if} i \rightarrow j \notin E(G). \end{array}\right. $$

## Introduction to Cyclic Subspaces and Connection to Frobenius Matrices

We will start with a motivational example of application of cyclic subspaces. We have a constant-recursive sequence

$$a_n = c_0 a_{n-1} + c_1 a_{n-2} + {\ldots} + c_{r-1} a_{n-r} $$

of order *r*, where all *c*_*i*_ are constants and initial conditions *a*_0_,…,*a*_*r*− 1_ are given. Perhaps, the most familiar example is the Fibonacci sequence *F*_*n*_ = *F*_*n*− 1_ + *F*_*n*− 2_.

We can define the *companion matrix* of our general sequence as:

$$C = \left[\begin{array}{llll} 0&\ldots&0 & -c_0\\ 1&\ddots& & -c_1\\ &\ddots& 0&{\vdots} \\ 0& & 1&-c_{r-1} \end{array}\right]. $$

The crucial property of the matrix *C* is that we can generate the next element of sequence with multiplication by *C*:

$$C^T \left[\begin{array}{lll} a_{n-r} \\ {\vdots} \\ a_{n-2} \\ a_{n-1} \end{array}\right] = \left[\begin{array}{lll} a_{n-r + 1} \\ {\vdots} \\ a_{n-1} \\ a_{n} \end{array}\right]. $$

For example, for Fibonacci sequence we have:

$$\left[\begin{array}{lll} 0 & 1 \\ 1 & 1 \end{array}\right] \left[\begin{array}{lll} F_k \\ F_{k + 1} \end{array}\right] = \left[\begin{array}{lll} F_{k + 1} \\ F_{k + 2} \end{array}\right]. $$

Now, if we want the *a*_*n*+ 1_ element of the series we can square the companion matrix and get:

$$C^T C^T \left[\begin{array}{lll} a_{n-r} \\ {\vdots} \\ a_{n-2} \\ a_{n-1} \end{array}\right] = C^T \left[\begin{array}{lll} a_{n-r + 1} \\ {\vdots} \\ a_{n-1} \\ a_{n} \end{array}\right] = \left[\begin{array}{lll} a_{n-r + 2} \\ {\vdots} \\ a_{n} \\ a_{n + 1} \end{array}\right]. $$

And analogously to get the *a*_*n* + *k*− 1_ element we need to compute the *k*-th power of the matrix *C*. In linear algebra such transformations are known as the *cyclic subspaces* generated by the vector *a*. For our purposes, we will restrict ourself to a conclusion, that some columns of a companion matrix occur in its powers. These properties are well known in the linear algebra theory (see [10, 13] for more cyclic properties).

### Consequences of Frobenius Normal Form

Let $\mathbb {F}$ be a commutative field. For any matrix $A \in \mathbb {F}^{n\times n}$ there exists an invertible *U* over $\mathbb {F}$ such that:

$$U^{-1} A U = F = \left[\begin{array}{lllll} C_1 & & & & 0 \\ & C_2 & & \\ & & C_3 & & \\ & & & {\ddots} & \\ 0 & & & & C_k \end{array}\right]. $$

and *F* is the Frobenius-canonical-form^2^ of *A*. The diagonal block *C*_*i*_ is called the companion matrix:

$$C_i = \left[\begin{array}{lllllll} 0& &{\ldots} & &0 & -c_0\\ 1&0& & &0 & -c_1\\ &1&{\ddots} & &{\vdots} & -c_2\\ & &{\ddots} &0& & {\vdots} \\ & & &1&0 & -c_{r-2}\\ 0& & & &1 & -c_{r-1} \end{array}\right] \in \mathbb{F}^{r\times r} . $$

Each companion matrix corresponds to the monic polynomial $C_{i}(x) = x^{r} + c_{r-1}x^{r-1} + {\ldots } + c_{0} \in \mathbb {F}\left [x\right ]$ (similarly to the sequence example) and this polynomial is called the *minimal polynomial* of *A*. Each minimal polynomial has a property that *C*_*i*_(*A*) = 0. To guarantee that matrix has only one decomposition into Frobenius normal form we require that every polynomial must divide the next one, i.e., *C*_*i*_(*x*)|*C*_*i*+ 1_(*x*). The final list of polynomials is called the *invariant factors* of matrix *A* [25]. Storjohann [25] proposed the deterministic algorithm to compute the Frobenius canonical-form efficiently.

#### **Theorem 2**

[25] *The Frobenius canonical-form of a matrix can be computeddeterministically using*$\widetilde {O}(n^{\omega })$*fieldoperations.*

Moreover, there are also probabilistic algorithms that compute this form in expected $\widetilde {O}(n^{\omega })$ time over small fields [10]. In this paper, all algorithms are deterministic if we the upper bound on the number of distinct walks is *W*. Then, due to the time of a single field operation we need additional *O*(log *W*) factor in the complexity. However, since we are mainly interested in determining if a cycle/walk of a given length exists in a graph, we can set a sufficiently small field $\mathbb {Z}_{p}$ (*p* has *O*(log *n*) bits). This way when algorithm returns nonzero we are sure that there exists some walk. If algorithm returns zero, then with high probability there will be no such walk.

### Cyclic Subspaces

Frobenius decomposition can be used to get the desired power of a matrix (analogously to the diagonal decomposition):

$$A^k = (U F U^{-1})^k = U F (U^{-1} U) F {\cdots} F (U^{-1} U) F U^{-1} = U F^k U^{-1}. $$

Moreover, we will use the property that the power of block diagonal matrix *F* is block diagonal:

$$F^k = \left[\begin{array}{lllll} C_1^k & & & & 0 \\ & C_2^k & & \\ & & C_3^k & & \\ & & & {\ddots} & \\ 0 & & & & C_l^k \end{array}\right] .$$

**Fig. 1:**
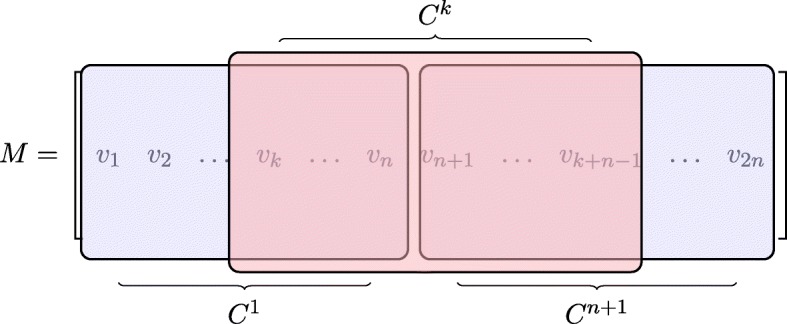
Visualisation of the cyclic property (Definition 1)

Now, we need a property of companion matrices that will enable us to power them efficiently (Fig. 1).

#### **Definition 1** (Cyclic Property)

Let *v*_1_,…,*v*_*n*_ be the columns of a matrix $C \in \mathbb {F}^{n\times n}$. Let *v*_*n*+ 1_,…,*v*_2*n*_ be the columns of matrix *C*^*n*+ 1^. If, for every 1 ≤ *k* ≤ *n* the columns of matrix *C*^*k*^ are *v*_*k*_,*v*_*k*+ 1_,…,*v*_*k* + *n*_ then the C has a **cyclic property**.

It turns out, that companion matrices have a cyclic property.

#### **Theorem 3** (Folklore 13, see [17] for generalization)

*Every companion matrix has a cyclic property.*

We will illustrate this property with an example:

$$C = \left[\begin{array}{lllll} 0 & 0 & 0 & 0 & 1 \\ 1 & 0 & 0 & 0 & 2 \\ 0 & 1 & 0 & 0 & 3 \\ 0 & 0 & 1 & 0 & 4 \\ 0 & 0 & 0 & 1 & 5 \end{array}\right], C^{2} = \left[\begin{array}{lllll} 0 & 0 & 0 & 1 & 5 \\ 0 & 0 & 0 & 2 & 11 \\ 1 & 0 & 0 & 3 & 17 \\ 0 & 1 & 0 & 4 & 23 \\ 0 & 0 & 1 & 5 & 29 \end{array}\right], C^{3} = \left[\begin{array}{lllll} 0 & 0 & 1 & 5 & 29 \\ 0 & 0 & 2 & 11 & 63 \\ 0 & 0 & 3 & 17 & 98 \\ 1 & 0 & 4 & 23 & 133 \\ 0 & 1 & 5 & 29 & 168 \end{array}\right], $$

$$C^{4} = \left[\begin{array}{lllll} 0 & 1 & 5 & 29 & 168 \\ 0 & 2 & 11& 63& 365 \\ 0 & 3 & 17& 98& 567 \\ 0 & 4 & 23& 13& 770 \\ 1 & 5 & 29& 16& 973 \end{array}\right], C^{5} = \left[\begin{array}{lllll} 1 & 5 & 29 & 168 & 973 \\ 2 & 11 & 63 & 365 & 2114 \\ 3 & 17 & 98 & 567 & 3284 \\ 4 & 23 & 133 & 770 & 4459 \\ 5 & 29 & 168 & 973 & 5635 \end{array}\right] C^{6} = \left[\begin{array}{lllll} 5 & 29 & 168 & 973 & 5636 \\ 11 & 63 & 365 & 2114 & 12243 \\ 17 & 98 & 567 & 3284 & 19019 \\ 23 & 133 & 770 & 4459 & 25824 \\ 29 & 168 & 973 & 5635 & 32634 \end{array}\right] $$

The matrix *C*^*i*^ has 4 columns identical to matrix *C*^*i*+ 1^. *C* has coefficients of order equal to dimension (dimension is 5 and maximum coefficient is 5). After powering to the 5th power, the coefficients can be of order 5^5^. Over a finite field $\mathbb {Z}_{p}$, all those coefficients will have *O*(log *p*) bits.

## Matching Distance Queries on Directed Unweighted Graphs

In this section, we will present an algorithm that matches the best known upper bounds of [33] for distance queries in directed unweighted graphs and uses Frobenius matrices.

We take the adjacency matrix *A* of a graph *G* (i.e., *n* × *n* matrix with *a*_*u*, *v*_ = 1 when (*u*, *v*) ∈ *G* and 0 otherwise). The *k*-th power of the adjacency matrix of the graph *G* holds the number of walks, i.e., an *a*_*u*, *v*_ element of *A*^*k*^ is *the count of distinct walks fromutovof lengthkin the graph*.

### **Observation 1** (Folklore, [8])

Let *A* ∈{0,1}^*n*×*n*^ be the adjacency matrix of a directed graph G. The (*A*^*k*^)_*u*, *v*_ is the number of distinct walks from u to v of length exactly k in the graph G.

Hence, the shortest path between vertices *u*, *v* is the smallest *k* such that *A*^*k*^ has nonzero element *a*_*u*, *v*_. This will allow us to forget about graph theory interpretation for a brief moment and focus only on finding such *k* with algebraic tools.

In this section we will proof the following Lemma.

### **Lemma 1**

*Given a matrix*$A \in \mathbb {F}^{n \times n}$*.There exists an algorithm that after some preprocessing, can answer queries for any givenpair of indicesi*, *j* ∈{1,…,*n*} *and integerk* ∈{1,…,*n*}, *such that:*

- *query returns an element* (*A*^*k*^)_*i*, *j*_,
- *preprocessing takes*$\widetilde {O}(n^{\omega })$*fieldoperations and query takesO*(*n*) *field operations.*

*The algorithm is deterministic.*

To proof this Lemma we decompose matrix *A* into the Frobenius normal form. Storjohann [25] showed an algorithm that returns *U* and *F* deterministically in $\widetilde {O}(n^{\omega })$ field operations (note that matrix inverse can also be computed in $\widetilde {O}(n^{\omega })$ field operations).

To better explain the idea, in the next section we will consider a simple case when a number of invariant factors of *A* is exactly 1. Then in Section 4.2 we will show how to generalize it to multiple invariant factors.

### Single Invariant Factor

In that situation, the matrix *F* is a companion matrix $C \in \mathbb {F}^{n\times n}$. First, we compute the (*n* + 1)-th power of the companion matrix *F*^*n*+ 1^. This can be done by using $\widetilde {O}(n^{\omega })$ field operations by repeated squaring (compute *F*, *F*^2^,*F*^4^,…,*F*^*n*+ 1^ with *O*(log *n*) matrix multiplications).

**Fig. 2:**
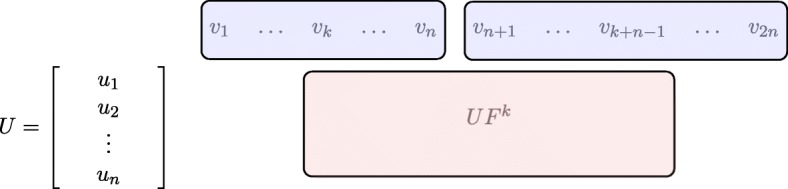
Construction of *UF*^*k*^ from matrices *UF* and *UF*^*n*+ 1^

Let *v*_1_,…,*v*_*n*_ be the columns of matrix *UF* and *v*_*n*+ 1_,…,*v*_2*n*_ be the columns of matrix *UF*^*n*+ 1^. Note, that because matrix *F* has a cyclic property, the columns *v*_*k*_,…,*v*_*k* + *n*− 1_ construct *UF*^*k*^ (see Fig. 2).

This step took just two matrix multiplications, because we need to multiply *U* times *F* and *F*^*n*+ 1^. The preprocessing phase takes only $\widetilde {O}(n^{\omega })$ field operations.

Now, if a query asks for the number of distinct walks from vertices *u* to *v* of length exactly *k* we:

- select *u*-th row of matrix *UF*^*k*^ (*n* numbers),
- select *v*-th column of matrix *U*^− 1^,
- multiply them in by using *O*(*n*) multiplications (dot product of two *n*-dimensional vectors).

This will give us the *u*, *v* element of matrix *UF*^*k*^*U*^− 1^ = *A*^*k*^. To get the length of the shortest path (i.e., the minimal *k* such that (*A*^*k*^)_*u*, *v*_ > 0), we will modify our matrices slightly to get the number of walks of length ≤ *k*. At the end, we will use in $\widetilde {O}(n)$ query tim (by using binary search) and $\widetilde {O}(n^{\omega })$ preprocessing time.

Basically, for a given *k* we need to get the *u*, *v* element of matrix *A* + *A*^2^ + ⋯ + *A*^*k*^. It suffices to add consecutive columns of matrix *UF* ⊕ *UF*^*n*+ 1^ = *v*_1_ ⊕ *v*_2_ ⊕… ⊕ *v*_2*n*_ in the following manner:^3^

$$M^{\prime} = \left[\begin{array}{ccccccc} v_1 & v_1 + v_2 & v_1 + v_2 + v_3 & {\ldots} & {\sum}^k_{i = 1} v_i & {\ldots} & {\sum}^{2n}_{i = 1} v_i \end{array}\right] \in \mathbb{F}^{n \times 2n} .$$

Now, to get *A* + *A*^2^ + ⋯ + *A*^*k*^ one would need to multiply $M^{\prime }_{k,k+n-1} U^{-1}$ and subtract $M^{\prime }_{1,n} U^{-1}$ for a balance.^4^

The naive algorithm can transform matrices *U* and *F* to matrix *M*^′^ in *O*(*n*^2^) field operations during preprocessing. During query, we will need to compute two dot products (*u*-th row of $M^{\prime }_{k,k+n-1}$ times *v*-th column of *U*^− 1^ and *u*-th row of $M^{\prime }_{1,n}$ times *v*-th column of *U*^− 1^) and subtract them.

We have an algorithm that for a given vertices *u*, *v* ∈ *G* and integer *k* ∈{1,…,*n*} can answer: *how many walks fromutovof length less or equalkare in the graphG* in $\widetilde {O}(n)$ query time and $\widetilde {O}(n^{\omega })$ preprocessing time.

Because the result of the query is increasing in *k* we can use binary search. We can determine the first *k* for which the query will answer nonzero value in *O*(log *n*) tries. Hence, in $\widetilde {O}(n)$ we can find the length of the shortest path. This generalized query can also return the number of walks of length exactly *k*, i.e., *q*(*u*, *v*, *k*) − *q*(*u*, *v*, *k* − 1).

We matched the result of [33] for unweighted graphs with a single invariant factor. In the next section, we will show how to generalize this technique for graphs with any number of invariant factors.

### Multiple Invariant Factors

Now, we will consider a case when *k* ≥ 1, i.e., matrix *F* has multiple invariant factors. First of all, we need to note that this generalization is not perfect and will allow to compute the number of walks of length up to *D* (the longest distance in a graph, i.e., diameter).

In a real world applications of our framework (detecting cycles, determining distance between nodes, etc.) one does not need to consider walks longer than the longest possible distance in a graph. It is natural that the diameter is considered to be a bound of an output in graph problems [1, 2, 7, 9].

#### Relation of the Graph Diameter and Frobenius Normal Form

We begin with relating the graph diameter to the Frobenius normal form. It turns out that the graph diameter is bounded by the degree of a smallest invariant factor.

##### **Lemma 2**

[8] *Given a directed, unweighted graph* G *with a diameter* D*. Letμdenote the degree of the smallest invariant factor (i.e.,the dimension of the smallest block in the Frobeniusmatrix* F*) of an adjacency matrix of the graph* G*. ThenD* ≤ *μ.*

This theorem is well known in literature [8]. We include the proof of this theorem for completeness.

##### Proof

For a contradiction assume that *D* > *μ* and let *u*, *v* ∈ *G* be vertices such that *δ*(*u*, *v*) = *D*. Let *A* be the adjacency matrix of *G*. We know, that there is the minimal polynomial of degree *μ*:


Term $a^{k}_{i,j}$ denotes the *i*, *j* element of the matrix *A*^*k*^. Now, consider the elements *u*, *v* of each matrix. The diameter *D* > *μ* and *δ*(*u*, *v*) = *D*, so for every *k* ≤ *μ* the elements $a^{k}_{u,v} = 0$ (because there is no walk of length less than *D* from *u* to *v*). Now, if we multiply the minimal polynomial by the matrix *A* we get:

$$A^{\mu+ 1} = a_0 A + a_1 A^2 + a_2 A^3 + {\ldots} + a_{\mu-1} A^{\mu} . $$

Hence $a^{\mu + 1}_{u,v} = 0$, because every element in the sequence $a^{k}_{u,v} = 0$ for *k* ≤ *μ*. By repeating this reasoning, we get that for every *k* > 0 the element $a^{k}_{u,v} = 0$. So, for every achievable pair of vertices, there must be some *k* ≤ *μ*, such that $a^{k}_{u,v} \ne 0$ and diameter is bounded by *μ*. □

The bounds of this inequality are tight. There are graphs with diameter *D* = *μ* and graphs with *μ* = *n* and arbitrary small diameter [8]. Our algorithms are able to return walks up to the length *μ*. We use the bound on *D* solely because it is easier to interpret diameter than the *smallest degree of the invariant factor*.

#### Generalization to Multiple Invariant Factors

**Fig. 3:**
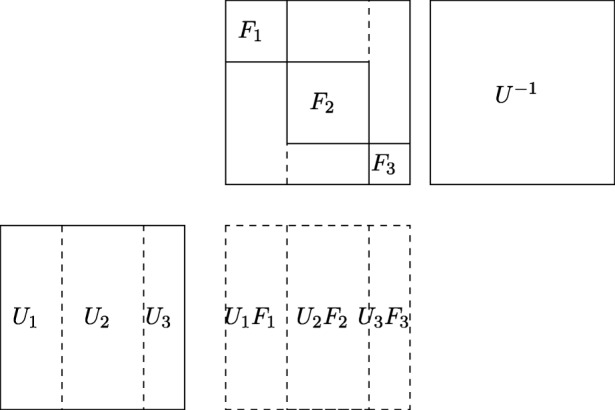
Multiplication of the *UFU*^− 1^. Example for 3 invariant factors. We know that matrix *F* is block and consists of *F*_1_,*F*_2_,*F*_3_. We divide matrix *U* into strips *U*_1_,*U*_2_,*U*_3_ that corespond to blocks of *F*. The observation is that we can compute *U*_1_ ⋅ *F*_1_, *U*_2_ ⋅ *F*_2_ and *U*_3_ ⋅ *F*_3_ independently and concatenate them into matrix *UF*

Let *k* denote the number of blocks in the Frobenius matrix *F* and *μ* be the number of columns of the smallest block. To multiply the matrix *U* by *F* we can start by multiplying strips of matrix *U* by appropriate blocks of *F* and concatenate them later (see Fig. 3).

We start by splitting the matrix *U* into *k* strips with rows corresponding to the appropriate blocks of *F* (strip *U*_*i*_ has as many columns as block *F*_*i*_). Then we multiply *UF* and have *k* strips: *U*_1_*F*_1_,*U*_2_*F*_2_,…*U*_*k*_*F*_*k*_ (each with at least *μ* columns). Next, we multiply *UF*^*μ*^ and also keep *k* strips: $U_{1} F_{1}^{\mu }, U_{2} F_{2}^{\mu },\ldots ,U_{k} F_{k}^{\mu }$. Our goal is to get a data structure such that if we need *UF*^*k*^, we can quickly choose appropriate columns and append them.

**Fig. 4:**
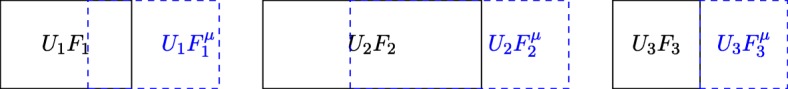
Combining strips into a single matrix. The height of the matrix in the schema is scalled down. We computed *U*_1_*F*_1_ and $U_{1} F_{1}^{\mu }$. Now we noted that companion matrices have a cyclic property so some of the rows in the strips are repeated. So in the single strip we can store only subsequent columns

The matrix *U*_*i*_*F*_*i*_ has *l*_*i*_ columns: $v_{1},\ldots , v_{l_{i}}$. Because *F*_*i*_ is a companion matrix, the $U_{i} F_{i}^{\mu }$ has the cyclic property (Definition 1). And the matrix $U_{i} F_{i}^{\mu }$ has columns: $v_{\mu },\ldots , v_{\mu +l_{i}}$. Note, that there are some duplicate columns in *U*_*i*_*F*_*i*_ and $U_{i} F_{i}^{\mu }$, when *μ* < *l*_*i*_. Hence, we only need to keep columns $v_{1},\ldots ,v_{\mu +l_{i}}$ for this strip. We do this for all strips *U*_1_*F*_1_,…*U*_*k*_*F*_*k*_ (see Fig. 4).

We are left with a matrix that has at most 2*n* columns (because $l_{1} + \mu + l_{2} + \mu + {\ldots } l_{k} + \mu = k \mu + {\sum }^{n}_{i = 1} l_{i} = n + k\mu \le 2n$). To generate it we need to power *F* to *μ* and do multiplications *U* ⋅ *F* and *U* ⋅ *F*^*μ*^. This can be computed in $\widetilde {O}(n^{\omega })$ field operations via fast matrix multiplication and repeated squaring.

#### Queries with Multiple Invariant Factors

**Fig. 5:**
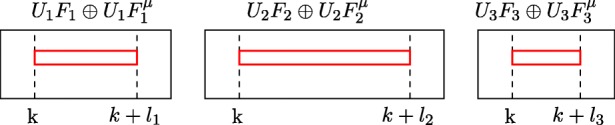
Schema of obtaining vector $\bar {u}$ (marked red) from 3 strips. We are given the row number and the power *k* and the lenghts *l*_*i*_ of each strip. At the end we concatenate them

When a query for the number of walks of length *k* from node *u* to *v* comes, we do:

1. For each strip *i* take the *u*-th row of $U_{i} F_{i} \oplus U_{i} F_{i}^{\mu }$ and concatenate them (see Fig. 5) into vector $\bar {u}$,
2. Take *v*-th column of *U*^− 1^ matrix and denote it $\bar {v}$,
3. Return the dot product $\bar {u} \cdot \bar {v}$.

Because *l*_1_ + *l*_2_ + … + *l*_*k*_ = *n* the vector $\bar {u} \in \mathbb {F}^{n}$. Query needs *O*(*n*) field operations.

Finally, this dot product is *a*_*u*, *v*_ element of the matrix *UF*^*k*^*U*^− 1^, for a fixed *k* ≤ *μ* because $\bar {u}$ is the concatenation of original vector *u*. Analogously to Section 4.1 one can extend this result to return the number of walks of length less or equal *k*. This matches (up to the polylogarithmic factor) the result of [33]. We will omit the details of this observation because in the next section we will extend this framework even further.

## Almost Optimal Query

In the previous section, we showed how to preprocess a matrix *A* with $\widetilde {O}(n^{\omega })$ field operations in such a way that in query that uses *O*(*n*) field operations we can return a number (*A*^*k*^)_*i*, *j*_. However, in linear time *O*(*n*) we return only a single number. The goal of this section is to get far richer information in $\widetilde {O}(n)$ query time (with extra factors from field operations).

### **Theorem 4**

*LetA* ∈{0,1}^*n*×*n*^*be a matrix such that the degree of smallest invariant factor isμ. Thereexists a deterministic algorithm that after some preprocessing can answer queries for anygiveni*, *j* ∈{1,…,*n*}*:*

- *Query returns* {*a*_*k*_|1 ≤ *k* ≤ *μ*},*wherea*_*k*_ = (*A*^*k*^)_*i*, *j*_ ,
- *Preprocessing takes* $\widetilde {O}(n^{\omega }\log {W})$ *and* *query takes* $\widetilde {O}(n\log {W})$ *time,*

*where* W *is an upper bound ona*_*k*_*for allk* ∈{1,…,*μ*}*.*

Note, that this theorem has some immediate application in graph algorithms (see Section 6).

### Hankel Matrix

Now, we will focus on the proof of Theorem 4. First, we need to introduce the Hankel matrix and its properties.

$$H = \left( \begin{array}{cccccc} c_1 & c_2 & {\ldots} & c_n \\ c_2 & c_3 & {\ldots} & c_{n + 1} \\ {\vdots} & {\vdots} & & {\vdots} \\ c_n & c_{n + 1} & {\ldots} & c_{2n-1} \end{array}\right) $$

Hankel matrix is defined by its first row and last column (2*n* − 1 numbers define *n* × *n* Hankel matrix). The numbers from the previous row are left-shifted by one and the new number is added at the end. Hankel matrices have some similarities to Topelitz and Circulant matrices.

The basic property we need is that the product of Hankel matrix and vector can be computed in *O*(*n* log *n*) time (see [15, 26]) even though explicitly writing the Hankel matrix as *n* × *n* matrix takes *O*(*n*^2^) time. The algorithm takes 2*n* − 1 parameters that define the Hankel matrix and *n* parameters that define the vector. The technique is based on the Fast Fourier Transformation [15, 26].

### Using Hankel Matrices to Get Richer Query

**Fig. 6:**
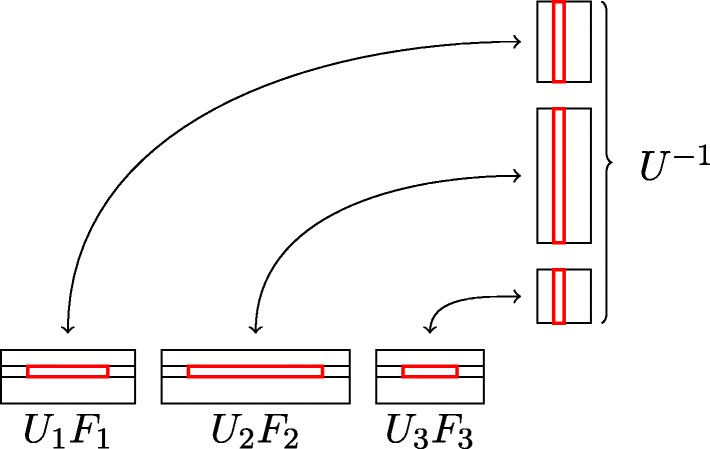
Multiplication of strips by *U*^− 1^ matrix. As you can see, matrix *U*^− 1^ can be splited into sections, that multiply only *U*_*i*_*F*_*i*_ strips

To proof the Theorem 4 we will modify only the last step in Section 4.2.3. The algorithm from Section 4.2.3 concatenates the strips *U*_*i*_*F*_*i*_ and builds a single vector. Subsequently, that vector is multiplied by a column of matrix *U*^− 1^. But we can also do it in a different order: first we multiply the strip by a section of matrix *U*^− 1^ and sum the results at the end. Thus, we perform *k* (number of strips) dot products of smaller vectors (see Fig. 6).

Consider the query for a number of walks of length exactly *k*. The strips in the matrix *U*^− 1^ do not depend on *k* (vector (*u*_0_,…,*u*_*l*_)). However, the vector taken from *U*_*i*_*F*_*i*_ (vectors (*x*_*i*_,…,*x*_*i* + *l*_)) will be left shifted if we want to compute the next one.

$$\begin{array}{ccccccccr} (x_0 & x_1 & x_2 & {\ldots} & x_l )\\ (x_1 & x_2 & {\ldots} & x_l & x_{l + 1} )\\ (x_2 & {\ldots} & x_l & x_{l + 1} & x_{l + 2})\\ {\vdots} & & & & {\vdots} \\ (x_{\mu} & &{\ldots} & & x_{\mu+l}) \end{array} \times \left( \begin{array}{cc}{u_0}\\{\vdots}\\{u_l} \end{array}\right) $$

As you can see, the subsequent rows can be written as the Hankel matrix (we need to add zeros to get a square matrix, but it will not influence asymptotic complexity since there will be at most *O*(*n*) of them). By using the *fast Hankel matrix-vector multiplication* we can compute *μ* values for every strip *i* in time *O*(*l*_*i*_ log *l*_*i*_) (*l*_*i*_ was defined as the length of *i*-th strip). At the end, we need to sum all results into a single array. Therefore, the number of operations is $O\left ({\sum }_{i = 1}^{k} l_{i} \log l_{i} \right )$. Because ${\sum }^{k}_{i = 1} l_{i} = n$ the algorithm needs *O*(*n* log *n*) field operations. This proves Theorem 4.

Here, we silently assumed that the number of walks is bounded by *W*. Note, that for large *W*, the algorithm needs to output *O*(*n*^2^ log *W*) bits and the complexity of every arithmetic operation needs to be multiplied by log *W*. If one is only interested in the deciding if an entry of some power of adjacency matrix is nonzero, we can use a standard randomization technique to eliminate log *W* factors from the running time.

#### **Corollary 1**

*LetA* ∈{0,1}^*n*×*n*^*be a matrix such that the degree of smallest invariant factor isμ. Thereexists an algorithm that after some preprocessing can answer queries for any giveni*, *j* ∈{1,…,*n*}*:*

- *Query returns* {*a*_*k*_|1 ≤ *k* ≤ *μ*},*wherea*_*k*_ = 1 *if* (*A*^*k*^)_*i*, *j*_*is nonzero,*
- *Preprocessing takes* $\widetilde {O}(n^{\omega })$ *and* *query takes* $\widetilde {O}(n)$ *time.*

*The algorithm is randomized with one-sided bounded error.*

#### Proof

At the beginning we will randomly select a prime number with *O*(log *n*) bits. We can write the matrix A as a polynomial:

$$\tilde{A}_{i,j} = \left\{\begin{array}{lll} x_{i,j} & \text{if} A_{i,j} = 1 \\ 0 & \text{otherwise}, \end{array}\right. $$

where *x*_*i*, *j*_ are unique variables. Now we can apply Schwartz-Zippel Lemma [23, 34]. The $\tilde {A}^{k}_{i,j}$ is the polynomial of degree at most *n* (because *k* ≤ *μ* ≤ *n*) and if we compute it over $\mathbb {Z}_{p}$ for *p* = *O*(*n*^2^) we can correctly determine if $\tilde {A}^{k}_{i,j}$ is a nonzero polynomial with a constant probability. We can repeat the above procedure *O*(log *n*) times to get a correct result in all entries with high probability. □

## Applications

In this section we will show how to use Theorem 4 to improve known algorithms on graphs. First we will develop a data structure that returns a number of distinct walks efficiently.

### **Lemma 3**

*LetG* = (*V*, *E*) *be a directed, unweighted graph with* n *vertices and a diameter* D*. There exists andeterministic algorithm that after some preprocessing can answer queries for any givenu*, *v* ∈ *V:*

- *Query returns* {*w*_*i*_|1 ≤ *i* ≤ *D*},*wherew*_*i*_*is the number of distinct walks from* u *to* v *of length exactly* i,
- *Preprocessing takes* $\widetilde {O}(n^{\omega }\log {W})$ *and* *query takes* $\widetilde {O}(n\log {W})$ *field* *operations.*

*whereWis the upper bound onw*_*i*_*for alli* ∈{1,…,*D*}.

### Proof

We encode the graph *G* as an adjacency matrix *A*(*G*). We use the Theorem 4 to construct the data structure that given a query (*u*, *v*) outputs (*A*^*k*^)_*u*, *v*_ for all 1 ≤ *k* ≤ *μ*. Finally, we use Observation 1 to note, that (*A*^*k*^)_*u*, *v*_ is equal to the number of distinct walks from u to v of length exactly k. Moreover we use Lemma 2 to bound the number *D* ≤ *μ*, so we will always output more numbers (but we can truncate them in *O*(*n*) time). Finally we note, that the preprocessing and query of Theorem 4 matches the statement and construction of adjacency matrix is *O*(*n*^2^). □

This algorithm is a significant improvement over [33]:

- One can use Lemma 3 to find the distance between *u*, *v* by linearly scanning the array and returning the first *k* such that *w*_*k*_ > 0,
- Lemma 3 can count cycles. In contrast the [33] cannot, because the distance from *u* to *u* is always 0 (see Section 6.1),
- Lemma 3 is almost optimal, i.e., when *D* = *O*(*n*) then query will need to output *O*(*n* log *W*) bits.

From the other hand, Lemma 3 is merely a functional improvement and it does not break the $\widetilde {O}(n^{\omega })$ of the *Single Source Shortest Paths* (SSSP) for dense, directed graphs.

Now we will show the application of Lemma 3. We begin with almost optimal algorithm to compute the number of all walks between all pairs of vertices. We are not aware of any other research concerning this problem.

### **Definition 2** (All-Pairs All Walk problem)

Given a directed, unweighted graph *G* with a diameter *D*. The task is to return an array *A*, such that for every pair of vertices *u*, *v* ∈ *G* and every *k* ∈{1,…,*D*} an element *A*[*u*, *v*, *k*] is the number of distinct walks from *u* to *v* of length *k*.

The folklore solution to this needs *O*(*Dn*^*ω*^ log *W*) time (where *W* is an upper bound on number of walks) and works as follows: *take the adjacency matrixAof graphGand save it inA*[*u*, *v*, 1]. *Then, square it to getA*^2^*and save it inA*[*u*, *v*, 2]. *Continue until you fill out complete table*. In the worst case this algorithm needs *D* = *O*(*n*) matrix multiplications, thus it needs *O*(*Dn*^*ω*^) field operations. At the first glance it is surprising that we can improve it to $\widetilde {O}(n^{3})$ field operations.

### **Theorem 5**

*All-Pairs All Walk problem admits an*$\widetilde {O}(n^{3} \log {W})$*algorithm(where* W *is upper bound on number of walks between every pair of vertices).*

### Proof

We will apply the Lemma 3 algorithm. The preprocessing takes $\widetilde {O}(n^{\omega })$ time. Then, for every pair of vertices *u*, *v* ask a query. A single query takes $\widetilde {O}(n \log {W})$ time. Next we will save it in the table *A*[*u*, *v*] (query gives D numbers *w*_1_,…,*w*_*D*_, such that *w*_*i*_ is the number of walks of length i and save it *A*[*u*, *v*, *i*] := *w*_*i*_).

Because there are *O*(*n*^2^) pairs and for each pair we need $\widetilde {O}(n\log {W})$ time, the complexity of our solution is $\widetilde {O}(n^{3}\log {W})$. The algorithm is almost optimal because the output in the worst case may be *O*(*n*^3^ log *W*) (we may need *O*(log *W*) bits to encode a single entry in the table). □

### Counting and Determining the Lengths of Cycles

We will use Theorem 4 to solve All-Nodes Shortest Cycle (ANSC) problem efficiently.

#### **Definition 3** (All-Nodes Shortest Cycles 31)

Given a directed, unweighted graph *G*. The problem *All-Nodes ShortestCycle* asks to output for every vertex *v* the length of the shortest cycle that contains *v*.

#### **Lemma 4**

*There exists a deterministic algorithm that for a given unweighted, directed* *G* *with a diameter* *D:*

- *For every vertexureturnsDnumbers:*${c_{u}^{1}}, {c_{u}^{2}}, \ldots {c_{u}^{D}}$,*where*${c_{u}^{k}}$*isthe number of non-simple cycles of length exactlyk, that contain vertex u*,
- *Algorithm works in* $\widetilde {O}(n^{\omega } \log {W})$ *time* *(where* *W* *is an upper bound on* ${c^{k}_{u}}$ *).*

#### Proof

We will use Theorem 4. We start by preprocessing the graph *G* in time $\widetilde {O}(n^{\omega }\log {W})$. Theorem 4 allows us to ask for a number of walks from *u* to *v* and receive *D* numbers: $w_{u,v}^{k}$. So, we ask for the number of walks from vertex *u* to the same vertex *u*. This is exactly the number of non-simple cycles of given length that contain vertex *u*.

Because we need to ask only *n* queries (it is the number of vertices in a graph) and each query takes $\widetilde {O}(n\log {W})$ time we have $\widetilde {O}(n^{\omega }\log {W} + n^{2}\log {W})$ = $\widetilde {O}(n^{\omega }\log {W})$ algorithm. □

If we are only interested in deciding if the numbers ${c_{u}^{i}}$ are nonzero, instead of Theorem 4 we can use Corollary 1. It introduces the one-sided randomization but allows us to shave log *W* factors in the running time.

#### **Corollary 2**

*There exists a randomized algorithm that for a given unweighted, directed* *G* *with* *a diameter* *D* *:*

- *For every vertexureturnsDnumbers:*${c_{u}^{1}}, {c_{u}^{2}}, \ldots {c_{u}^{D}}$, *where*${c_{u}^{k}}$*is1 if there exists a non-simple cycle of length exactlyk, that contain vertex uor 0 otherwise,*
- *Algorithm works in* $\widetilde {O}(n^{\omega })$ *time* *with one sided bounded error.*

Now we will show how to improve upon [31] $\widetilde {O}(n^{(\omega + 3)/2})$ algorithm with Corollary 2.

#### **Theorem 6**

*All-Nodes Shortest Cycles admits an* $\widetilde {O}(n^{\omega })$ *randomized* *time algorithm.*

#### Proof

We use Lemma 2 to compute the table *S*[*v*]. For every vertex we search for the first nonzero element linearly. This with high probability is the length of the shortest cycle that contains it. Because the output contains *O*(*n*^2^) numbers the complexity is equal to the preprocessing time $\widetilde {O}(n^{\omega })$. □

Also the Corollary 2 improves upon [9, Theorem 45] for unweighted graphs.

#### **Corollary 3**

*Given a directed, unweighted graph* G *with a diameter* D*. LetS*(*c*) *denote the set of vertices that lie in the cycle of length exactly* c*.In*$\widetilde {O}(n^{\omega })$*timewe can return the setsS*(1),…,*S*(*D*) *with constant probability of success.*

#### Proof

Similarly to the proof of Theorem 6, we can scan the output to compute the set *S*(*c*) that contains all vertices that lie on some cycle of length ≤ *c*. Then, by linear scan we can return the sets *S*(1),…,*S*(*D*). □

## Conclusion and Open Problems

We introduced the framework based on Frobenius normal form and used it to solve some problems on directed, unweighted graphs in matrix multiplication time. The main open question is to use this framework to prove that APSP on such graphs can be solved in $\widetilde {O}(n^{\omega })$ or at least *O*(*n*^2.5^). The promising way is to use the algorithms that determine operators of matrices of polynomials (e.g., determinant, solving linear system [14, 18]). Additionally, algorithms for a black-box polynomial degree determination seem to be a promising path.

Another interesting problem is to use this framework to obtain additive approximation for APSP. Currently, the best additive approximation of APSP is due to [20]. However, no additive approximation of APSP is known that would work in $\widetilde {O}(n^{\omega })$ time.

Application in dynamic algorithm also seems to be a promising approach. Frandsen and Sankowski [12] showed an algorithm, that dynamically preserves Frobenius normal form in *O*(*kn*^2^) time. Our algorithms use fast Hankel matrix-vector multiplication that is based on FFT. Reif and Tate [19] presented an $O(\sqrt {n})$ time per request algorithm for FFT. Can we use these approaches to obtain a faster dynamic algorithm?

Finally, it remains open how to apply the Frobenius normal form in the weighted directed graphs with small, integer weights {−*M*,…,*M*}. Cygan et al. [9] took degree *M* polynomials and used [18] algorithms to get $\widetilde {O}(M n^{\omega })$ time radius and diameter detection. We suspect that similar technique can be applied to Frobenius normal form framework.

## Footnotes

1 Tr [*A*] denotes the trace of a matrix *A*, i.e., the sum of elements on the main diagonal.

2 Sometimes this form is called rational-canonical form.

3 Operation ⊕ denotes concatenation.

4 *X*_*a*, *b*_ denotes a matrix constructed by concatenating columns *x*_*a*_,*x*_*a*+ 1_,…,*x*_*b*_ of a matrix *X*.
